# Editorial: Sensory-driven neural circuit maturation and implications for neurodevelopmental disorders

**DOI:** 10.3389/fnins.2026.1977319

**Published:** 2026-09-22

**Authors:** Bor-Shuen Wang, Dhananjay Huilgol, Anirban Paul, Hillary C. Schiff

**Affiliations:** 1 Biology Department, St. Joseph's University, Brooklyn, NY, United States; 2 BRIC-National Brain Research Centre, Gurugram, India; 3 Penn State College of Medicine, Hershey, PA, United States; 4 Division of Biosciences, College of Dentistry, Ohio State University, Columbus, OH, United States

**Keywords:** critical period, development, neural circuit, neurodevelopmental disorders, sensory

How does experience shape biology? The developing brain is not simply assembled by genetic programs; it is progressively instructed by activity, first spontaneous, then sensory-driven, as connections are stabilized, remodeled, or lost. Classic studies of visual critical periods established this principle decades ago. Its reach now extends much further, from the cellular and molecular structures that anchor mature circuits in place (Ackerman et al., 2025; Ribot et al., 2025; Gibel-Russo et al., 2022), to the association and limbic circuits that never receive sensory input directly (Sydnor, 2026; Chen et al., 2025).

This window of developmental plasticity is also a window of vulnerability. Sensory disturbances are among the earliest detectable features of autism spectrum disorder, Angelman syndrome, Fragile X syndrome, and CDKL5 deficiency disorder, suggesting they reflect disrupted circuit maturation rather than isolated deficits (Shang and Zhang, 2025). Yet questions remain unresolved. How genetic disruption affects pre-sensory activity, how transient activity leads to lasting structural changes, and whether the sensitive windows characterized in the primary sensory cortex extend into the association and limbic circuits remain open questions.

The five articles in this Research Topic cover this broader view, each focusing on different aspects. Two examine different developmental stages within the visual pathway: spontaneous, pre-sensory activity itself (Gupta et al.), and the structural maturation that unfolds during the classical postnatal critical period (Emery et al.); a third extends the underlying mechanism to glia, a cell type largely absent from the classical account (Miller and Broadie); and the remaining two extend the anatomy entirely, to prefrontal and amygdalar circuits receiving no direct sensory input (Anqueira-González and Canetta; Saxon and Corbin).

Activity-dependent development begins even before external sensory experience arrives. Gupta et al. show that spontaneous retinal waves prior to eye opening are disrupted in a mouse model of Angelman syndrome. Loss of the maternal *Ube3a* allele reduces retinal ganglion cell firing and wave frequency without affecting wave propagation. This dissociation points to a deficit in wave initiation rather than in the circuitry that maintains activity, suggesting that pre-sensory activity patterns can be an early site of developmental disruption.

As development proceeds, external sensory experience refines these circuits. Emery et al. demonstrate this in a mouse model of CDKL5 deficiency disorder, mapping perineuronal net (PNN) expression across the primary visual cortex. Null mice show an early increase in PNNs across all visual cortex, followed by subregion-specific divergence at the peak of the critical period. This suggests CDKL5 loss disrupts the transition from plastic to mature circuitry rather than producing uniform abnormality.

Gupta et al. and Emery et al. trace two developmental epochs in the same visual pathway, and both show disruption as a selective phenomenon, rather than global. *Ube3a* loss impairs wave initiation while sparing the machinery that propagates each wave, whereas CDKL5 loss instead divides by region, pushing neighboring visual subregions in opposite directions. Even within a single pathway, a gene's disruption can be remarkably precise.

Miller and Broadie review a candidate mechanism for how activity results in lasting structural change, drawn from the *Drosophila* olfactory circuit, where glia act as serotonergic effectors of experience-dependent remodeling. In this relay, sensory experience drives phagocytic glia to upregulate serotonin synthesis, signaling a second glial class to clear the extracellular matrix that otherwise blocks access to synapses marked for pruning. This pathway is necessary for experience-dependent pruning in the olfactory circuit, and is implicated more broadly in Fragile X syndrome, where failure to prune contributes to lifelong impairments. Reactivating the same signaling in adulthood can reopen critical-period-like plasticity, pointing to a potential therapeutic strategy with implications in injury induced regeneration. Critical period research has traditionally centered on neurons, but a relay like this suggests some central questions may need to be asked of non-neuronal cell-types, increasingly being shown to be important in developmental plasticity.

Sensory-dependent development extends beyond primary sensory brain regions. Anqueira-González and Canetta review how early sensory and multisensory experience shapes prefrontal cortex (PFC) development, despite receiving no direct sensory input. Early sensory restriction and environmental enrichment produce opposing effects on PFC interneurons and myelination, with enrichment further enhancing PFC-dependent behaviors. Early life stress, trauma, and social isolation alter PFC structure and function in both humans and animals, affecting inhibitory interneurons, PNNs, glia, and dopamine signaling depending on the specific experience.

Saxon and Corbin extend this framework to socio-emotional circuitry in the amygdala, where two adjacent nuclei mature on markedly different timelines. The basolateral amygdala (BLA) matures within the first post-natal month and supports fear conditioning from the second post-natal week. As pups approach weaning, the BLA's role shifts from caregiver-dependent affiliation toward threat and novelty discrimination. However, the neighboring paralaminar nucleus remains immature well into adolescence and acquires responsiveness to novel cues only in adulthood. Early-life stress increases BLA dendritic complexity and PNN density but impairs BLA-PFC connectivity. Limited evidence suggests the paralaminar nucleus may be similarly sensitive to early deprivation, though less studied than the BLA.

Together, these two reviews find sensitive windows harder to pin down outside the primary sensory cortices. PFC studies rely on broad, overlapping age ranges, leaving it unclear whether discrete windows exist at all, or whether plasticity here is simply more gradual (Anqueira-González and Canetta). A related asymmetry appears in the amygdala: the basolateral nucleus is well-characterized within the first post-natal month, while the neighboring paralaminar nucleus, maturing far more slowly, remains largely unstudied (Saxon and Corbin). This distinction matters for intervention: primary sensory cortex offers a defined window for timing treatment, but for higher-order circuits that may regulate complex cognitive and psychiatric functions, windows for intervention timing have not been identified.

As we continue to ask how experience shapes biology, the frontier shifts from cataloging deficits toward deciphering the rules of vulnerability. The challenge now is to determine how a circuit's unique architecture dictates its trajectory of disruption, how far these disturbances propagate across interconnected systems, and, most importantly, which aspects remain reversible. If early sensory and structural phenotypes can reveal circuit dysfunction before it is fully expressed in behavior, they offer far more than a window into pathogenesis. They present a profound opportunity to intercept and redirect that trajectory while the developing brain remains plastic.
